# Transient Right Bundle Branch Block Resulting From a Blunt Cardiac Injury During a Motor Vehicle Accident

**DOI:** 10.7759/cureus.10534

**Published:** 2020-09-18

**Authors:** Nicholas L Biondi, Manoj Bhandari, Poonam Bhyan

**Affiliations:** 1 Internal Medicine, Campbell University School of Osteopathic Medicine, Buies Creek, USA; 2 Internal Medicine, Cape Fear Valley Medical Center, Fayetteville, USA; 3 Cardiolgy, Cape Fear Valley Medical Center, Fayetteville, USA

**Keywords:** blunt chest trauma, cardiac contusion, right bundle branch block, adult cardiology, general internal medicine, blunt cardiac injury, arrhythmia, cardiac trauma, medical education

## Abstract

Blunt chest trauma (BCT) has become increasingly more prevalent in recent years. As a result, the incidence of blunt cardiac injury (BCI), or cardiac or myocardial contusion, has also increased. The sequelae of BCI often are undiagnosed due to variability in the clinical presentation. This case highlights a transient right bundle branch block (RBBB) following a motor vehicle accident (MVA), resulting in BCI. Right-sided cardiac injuries predominate BCI owing to the anterior location of the right ventricle within the thoracic cage; however, the pathophysiologic mechanisms underlying the electrocardiographic manifestations are vaguely understood. In this case, a 66-year-old female sustained a BCI resulting in a transient RBBB. The patient fully recovered following a three-day hospitalization with complete recovery of normal cardiac conduction.

## Introduction

Traumatic injuries, especially myocardial injuries, are hallmarked by high morbidity and mortality and are the leading cause of death worldwide to the tune of 5.8 million fatalities annually [1]. Many of these traumatic injuries result in blunt chest trauma (BCT) and blunt cardiac injuries (BCI) [1-3]. The identification of these lesions dates back to the 1940s [4]. More than 60% of patients sustaining BCI will have an identifiable electrocardiogram (ECG) abnormality: T wave, ST or QT segments, or atrioventricular or ventricular conduction pathway abnormalities [1-3,5]. Sinus tachycardia is the most common arrhythmia following trauma, and atrial fibrillation is the next most common rhythm identified [3,5,6]. Transient phenomena consisting of premature ventricular contractions (PVC) and bundle branch blocks also occur with reproducible frequency with BCI having a classic association with a transient right bundle branch block (RBBB). Persistent RBBB has been reported but bears no clinical significance concerning patient outcomes and overall functionality [3].

Approximately half of BCI are attributed to high-impact mechanisms, such as motor vehicle accidents (MVA). Pedestrians being struck by motor vehicles, motorcycle accidents, falls, crush injuries, and direct chest traumas account for the majority of the remaining instances of BCI [1,3]. Many who sustain BCI have multiple other presenting injuries, but cardiac injuries are the most fatal. The most common injury is a transmural rupture of a cardiac chamber. Other resultant cardiac injuries from BCI include myocardial rupture, pericardial rupture, septal injury, valvular injury, myocardial infarction, myocardial contusion, and commotio cordis [1].

BCI is thought to develop from one of seven mechanisms [1,7].

1. Direct impact to the anterior chest wall, occurring when the ventricles are maximally distended in diastole.

2. Hydraulic pressure from a large abdominal force increasing cardiac pressures by raising preload pressures predisposing to rupture.

3. Compressive, bidirectional forces opposing the heart between the anterior sternum and posterior vertebral bodies.

4. Acceleration and deceleration forces, particularly in the anterior-posterior direction, leading to myocardial tears or lacerations and dissections of the epicardial, coronary arteries.

5. Direct, blast forces resulting in septal or ventricular rupture.

6. Concussive forces leading to cardiac arrhythmia.

7. Penetration and perforation of the myocardium by a displaced fracture.

## Case presentation

In July 2019, a previously well 66-year-old female presented to the emergency department (ED) following an MVA. The patient was a restrained driver during the collision with airbag deployment. She denied loss of consciousness during the accident. Immediately following impact, the patient reported acute onset abdominal pain, chest pain, dyspnea, and upper back pain.

The patient was previously well with a past medical history notable for benign essential hypertension, hypothyroidism, glucose intolerance, and chronic low back pain. She has undergone numerous non-cardiac and orthopedic surgical procedures and has no history of tobacco or illicit substance use. She has a significant family history of cardiac disease with early ischemic cardiomyopathy in her mother, father, sister, and bother.

**Figure 1:**
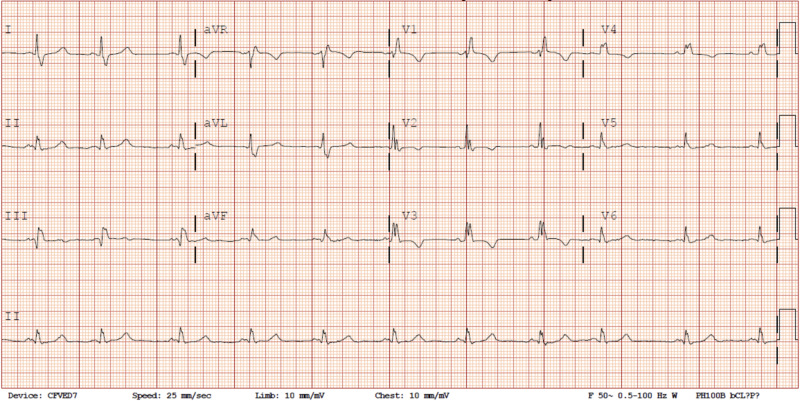
Presenting electrocardiogram following blunt chest trauma revealing a new right bundle branch block.

**Figure 2:**
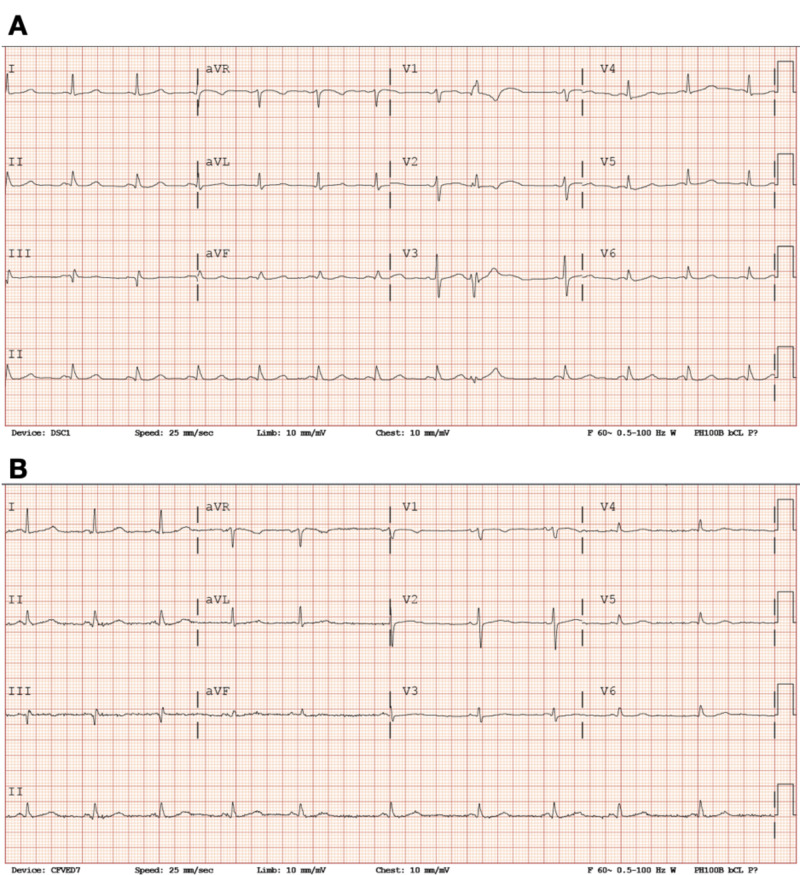
Previous electrocardiograms (A) Previous electrocardiograph from 2018 revealing sinus rhythm with the presence of a premature ventricular contraction. (B) Previous electrocardiograph from 2016 revealing normal sinus rhythm without conduction abnormality.

**Figure 3:**
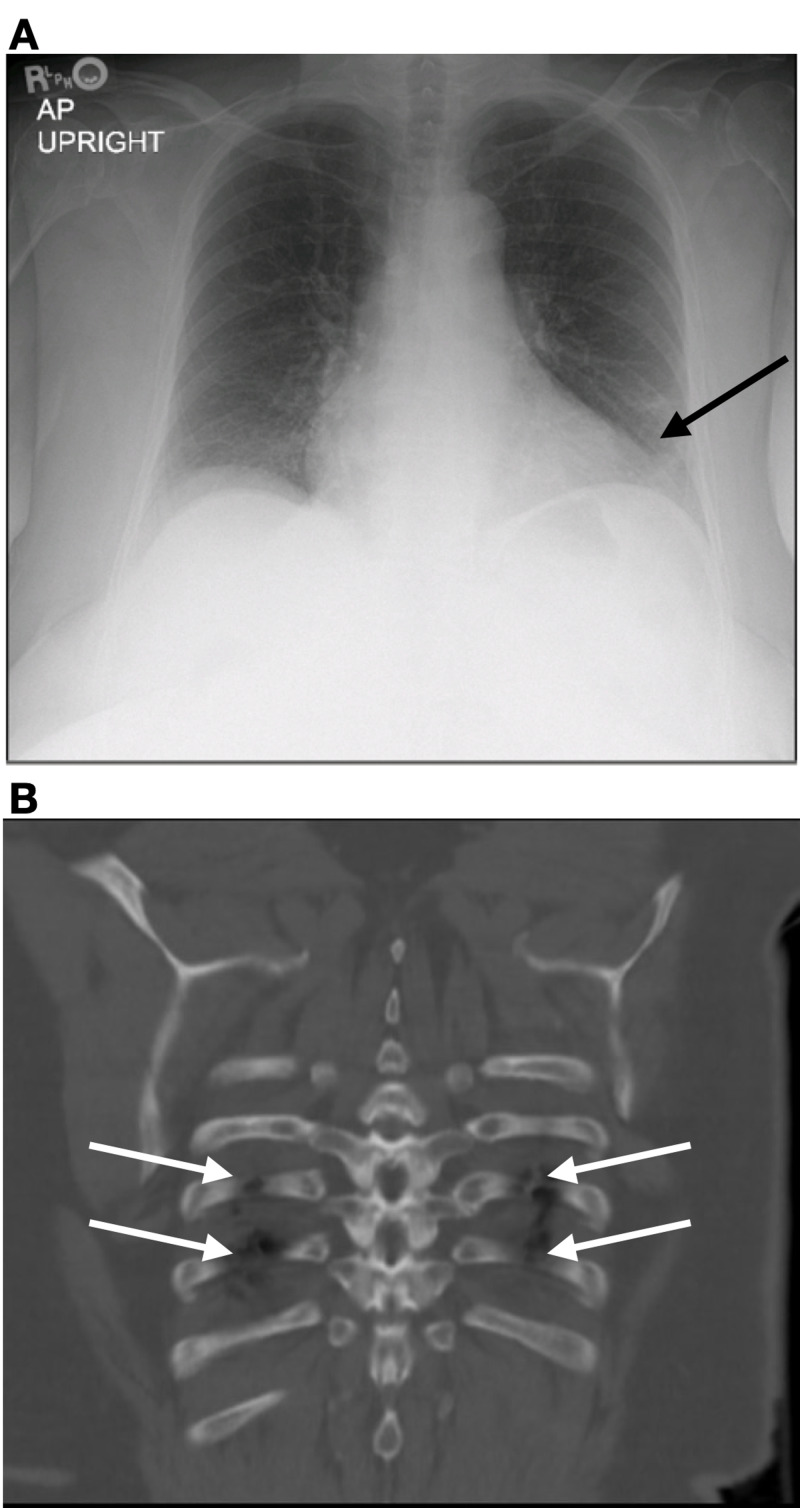
Presenting imaging (A) Anterior-posterior (AP), frontal chest radiograph. Findings: subsegmental atelectasis in the left lung base without acute cardiopulmonary pathology. (B) Computed tomography. Findings: multiple, anterior, rib fractures bilaterally without other organ damage.

Upon admission to the ED, the patient had a Glasgow Coma Scale (GCS) score of 15 and stable vital signs: blood pressure 152/73 mmHg, pulse 95 beats per minute, temperature 36.9°C (98.5°F), respiration rate 18 per minute, height 1.626 m (5' 4"), weight 96.6 kg (213 lb), and peripheral pulse oximetry (SpO_2_) 98% on room air. Physical exam revealed chest wall tenderness with anterior ecchymosis and sinus bradycardia. No extra heart sounds were auscultated, and the remainder of the physical exam was unremarkable. Presenting labs were notable for a mild leukocytosis without left shift, mild prerenal azotemia, and a detectable cardiac-specific troponin I, 0.078 ng/mL. Initial ECG (Figure 1) revealed a new RBBB. The patient had no history of prior cardiac conduction abnormalities (Figure 2). Chest radiograph was negative for significant abnormalities, but follow-up CT evaluation of the chest, abdomen, and pelvis revealed multiple, bilateral, anterior, rib fractures without underlying organ damage (Figure 3).

 

**Figure 4:**
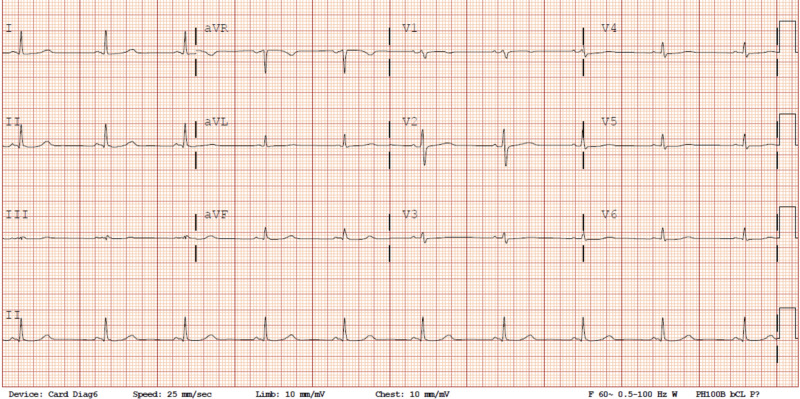
Repeat electrocardiogram from day 2 of the hospitalization showing resolution of the right bundle branch block conduction abnormality.

The patient was admitted for a suspected myocardial contusion. Repeat ECG on day 2 of the hospital stay (Figure 4) revealed resolution of the RBBB with the restoration of normal His-Purkinje conduction. The patient’s troponin peaked at 0.250 ng/mL (indeterminate range). Two-dimensional transthoracic echocardiography (TTE) showed normal biventricular function, normal wall motion, no pericardial effusion, and no significant valvular heart disease. The patient’s troponin returned to an undetectable level before discharge. The patient was managed conservatively during her stay and was discharged home on hospital day 3. She had an uneventful follow-up with her primary care provider five days post-discharge. 

## Discussion

The first case of transient RBBB following BCI was recorded in 1952 [8]. This recording may only serve as the first known occurrence of a condition that is likely more common than expected. Cardiac injury may represent the most common, unsuspected, visceral injury responsible for death in BCT and other traumatic injuries. Cardiac injuries are reported in a wide range of chest traumas from 6% to 76% [2,6]. Screening for this condition is commonplace including such modalities as ECG, TTE, and cardiac biomarkers [1,3]. Our patient was appropriately screened for BCI following her MVA with TTE, cardiac biomarkers, and ECG. The presence of a new-onset RBBB in the setting of detectable cardiac enzymes raised immediate suspicion for BCI via myocardial contusion.

The most common electrographic evidence of myocardial contusion seen in patients with BCI is ST depression, non-sustained ventricular tachycardia, varying degrees of heart block, and atrial arrhythmias. The ECG abnormalities are similar to myocardial ischemia. Histopathologic evaluation of contused myocardial tissue reveals intramyocardial hemorrhage, edema, and localized necrosis, which closely mimics myocardial infarction [1,6]. Slight differences between myocardial contusion and myocardial infarction include patchy areas of polymorphonuclear leukocyte infiltration and necrosis with abrupt transitions from contused tissue to normal myocardium. The hemorrhage is eventually resorbed with the formation of a myocardial scar. The areas of contusion are generally confined to specific myocardial bundles [6]. Despite these changes, the contusion is often of little clinical significance long term [9].

The right ventricle is the most anatomically anterior chamber of the heart. It is injured in 40% of myocardial contusion injuries followed by the right atrium and left ventricle, which account for 30%-33% of injuries [1,3]. BCT has stereotypical ECG abnormalities with ST-segment and T-wave abnormalities accounting for 35% of abnormalities [1-3,5,6]. Bundle branch conduction abnormalities account for approximately 10% of the ECG abnormalities seen with RBBB predominating in 90% of bundle branch block cases. RBBB predominates over left bundle branch block (LBBB) due to the anatomic location of the right ventricle [5].

Following BCI and myocardial trauma in general, the myocytes exhibit great irritability owing to ECG changes and arrhythmias. However, since the ECG is largely a reflection of the left ventricle due to its predominant mass and voltage-generating potential, the right ventricular injury may be masked thus limiting the sensitivity and specificity for detection of BCI [6]. A negative ECG carries a negative predictive value of approximately 95% for BCI in patients by BCT [1]. However, when combined with negative cardiac biomarkers, cardiac troponin less than 0.4 ng/mL, the negative predictive values approaches 100% [1,3,8,10].

The Eastern Association for the Surgery of Trauma (EAST) has authored screening and management guidelines for BCI. They have provided level I evidence for the use of admission ECG in all patients with suspected BCI. If this ECG returns abnormal, EAST provides level II evidence for continuous telemetric monitoring for 24-48 hours. Additional level II recommendations include obtaining further imaging studies such as TTE or transesophageal echocardiography (TEE) if the patient is hemodynamically unstable. Nuclear scans, however, are of little use in the evolution and management of BCI. A full listing of the EAST recommendations is shown in Table 1 [10].

**Table 1:** The Eastern Association for the Surgery of Trauma (EAST) guidelines for the evaluation and treatment of myocardial contusion. BCI, blunt cardiac injury; ECG, electrocardiogram; TEE, transesophageal echocardiography; TTE, transthoracic echocardiography

Level of Evidence	Recommendation
Level I	Admission ECG should be obtained in all patients with suspected BCI.
Level II	If admission ECG is abnormal, admit patient with continuous telemetry monitoring for 24-48 hours. If admission ECG is normal, further pursuit of diagnosis should be abandoned.
	If the patient is hemodynamically unstable, additional imaging (TTE or TEE) should be obtained.
	Nuclear medicine modalities provide little additional benefit compared with echocardiography and should be avoided if echocardiography has previously been performed.
Level III	Elderly patients with known cardiac disease, unstable patients, and those with abnormal admission ECGs are safe for operative intervention with close monitoring.
	The presence of a sternum is not predictive of the presence of BCI, and does not necessarily indicate the need for additional monitoring.
	Cardiac biomarkers (creatine kinase and troponin) are not useful in the prediction of complications related to BCI.

Cardiac contusion from BCI has been linked to circulatory system compromise and/or failure, dysrhythmias, and late rupture; often the consequences are less dramatic and clinically insignificant [6,9]. Presently, no definitive diagnostic criteria exist to define BCI. Screening procedures are in place in EDs across the world for prompt cardiac evaluation, but the most important part of the diagnostic algorithm remains a detailed history and physical examination [6].

This case demonstrates the transient nature of an RBBB following BCI with a cardiac contusion. The mechanism of injury, airbag deployment with rapid deceleration, fits the clinical context of BCI. Coupling the mechanism with ECG and serologic findings the diagnosis was made. The patient’s RBBB resolved by hospital day 2, and the patient was discharged home without incident. She required no intervention during her stay and had no lasting sequelae of the injury on routine outpatient follow-up.

## Conclusions

BCI, classically seen in the setting of trauma and non-penetrating injuries to the chest, is commonly complicated by lesions to the heart. A deliberate and thorough evaluation of cardiac injury remains an important first step in the evaluation of a BCI. Special attention should be paid to patients with abnormal ECGs and abnormal biomarkers in the setting of BCI. Presently, there is not a singular test to identify the presence of BCI with any clinical certainty. Clinical suspicion and adherence to the EAST guidelines will help providers establish a diagnosis and management plan for patients presenting with BCI. There is great clinical variability in the presentations and outcomes of BCI. Our case adds to the population of literature describing transient phenomena resulting from BCI. The initial transient RBBB resolved, and the patient sustained no lasting effects of her injury on post-hospitalization visits.
